# Severe Maternal Outcome in Women Admitted to an Obstetric Intensive Care Unit in the Northeast of Brazil: A Cross-Sectional Study

**DOI:** 10.1155/tswj/3559062

**Published:** 2025-04-29

**Authors:** Flávio Xavier da Silva, Ryta de Kássia Andrade Rufino, Micaelly Barbosa Padilha, Stephanie Karoline Santos Bezerra, Mario Diego Teles Correia, Leila Katz

**Affiliations:** ^1^Departamento de Medicina, Faculdade Pernambucana de Saúde (FPS), Recife, Pernambuco, Brazil; ^2^Centro de Atenção à Mulher (CAM), Instituto de Medicina Integral Prof. Fernando Figueira (IMIP), Recife, Pernambuco, Brazil

**Keywords:** critical care, high-risk pregnancy, maternal death, maternal mortality, maternal near-miss

## Abstract

**Objective:** The objective of this study is to describe the characteristics of women who experienced severe maternal outcomes (SMO: maternal near-miss or maternal death) in an obstetric intensive care unit (ICU).

**Methods:** A cross-sectional study was carried out including pregnant or postpartum women up to 42 days of childbirth admitted to the obstetric ICU at one reference centre in the northeast of Brazil, for any clinical, surgical, or obstetric complication, with data collected between October 29, 2018, and September 30, 2019. Maternal characteristics, details on admission to the ICU, pregnancy outcomes, and causes for ICU admission were compared between groups with SMO or with the remaining group, potential life-threatening conditions (PLTCs). A significance level of 5% was adopted.

**Results:** During the study period, 309 women were admitted to the obstetric ICU and considered eligible for the study. SMO was observed in 150 (48.5%) of these women. Of these, 8 (2.6%) were maternal deaths, and 142 (45.9%) presented one or more near-miss criteria. Most women with a SMO were admitted for direct obstetric causes such as hypertensive syndromes, postpartum haemorrhage, or puerperal infection. SMO was associated more frequently with puerperal infection.

**Conclusion:** SMO is a commonly occurring outcome in an obstetric ICU with great possibility of treatment. Direct obstetric causes such as hypertension syndromes, postpartum haemorrhage, and puerperal infection were the most prevalent causes in the development of this outcome. Puerperal infection was the condition most associated with SMO.

## 1. Introduction

Maternal near-miss and maternal death together, according to the World Health Organization (WHO), are considered as a severe maternal outcome (SMO) [1, 2]. The WHO refers to maternal morbidity as a continuum from minor to SMO, and on this continuum, there are potential life-threatening conditions (PLTCs) [3, 4]. Many maternal PLTCs are preventable and treatable. Postpartum haemorrhage, hypertensive syndromes, and infection are the most common causes of death, and when death is avoided, they can result in serious health injury [5–7].

Maternal death, according to the WHO [8, 9], is defined as the death of a woman while pregnant or within 42 days after childbirth, irrespective of duration or site of the pregnancy, due to any cause related to or aggravated by the pregnancy or its management, but not from unintentional or incidental causes. It is an important health indicator which reflects socioeconomic and ethnic deprivation of women, conditions that are difficult to change [10]. However, its prevention is extremely sensitive to obstetric care, and these may be changeable [11].

Additionally, other important definition is severe maternal morbidity (SMM) or “maternal near-miss” as the term recommended by WHO, which refers to a woman who survived very serious complications, during pregnancy, childbirth, or within a subsequent period of 42 days [12]. These women share many aspects with those who die, and they are in greater quantity, thus being an important predictor for the assessment of the quality of healthcare. There is no consensus on a single definition of SMM. However, it is based on clinical, intervention, and laboratory markers in accordance with the WHO [12].

Intensive care units (ICUs) represent an opportunity of protection for women who, in several cases, had their healthcare delayed: delay in deciding to seek care, delay in arriving at a health facility, and delay in the provision of adequate care [13]. There is evidence about their benefits in reducing maternal morbidity and mortality [14]. The advantages of an obstetric ICU within an obstetric setting are numerous, for instance, the concurrent availability of expert obstetric care and critical care management. Antenatal patients admitted to the obstetric ICU have the possibility of continuous fetal monitoring with on-hand expertise in its interpretation [15].

In the world, 287,000 maternal deaths were estimated for the year 2020, the first year of the COVID-19 pandemic, resulting in a maternal mortality ratio (MMR: maternal deaths per 100,000 live births (LBs)) of around 223 maternal deaths per 100,000 LBs [8]. The proportion of all COVID-19 deaths was uncertain [8]. The great majority of these deaths occurred in low-income countries as a result of complications related to pregnancy and childbirth [9]. High parity, poverty, difficult access to health facilities, and social and regional discrepancies have been some of the challenges faced by many countries in reducing maternal death [16, 17].

Maternal mortality (MM) in Brazil has not decreased enough in recent years. According to data available in the Brazilian information system about mortality (SIM), 1640 deaths of this nature occurred in the year 2020, which represents an MMR of 64 deaths per 100,000 LBs, almost five times higher than rich countries [18]. Of this total, 32% occurred in the northeast region [18]. These data will probably increase in coming years as a consequence of the pandemic caused by the SARS-CoV-2 virus, which in 2021 had already reached an alarming lethality rate of 7.2% in pregnant and postpartum women, more than double the lethality of the general population of the country (2.8%) [19].

This study described the characteristics of women who experienced SMO in accordance with the WHO criteria, in an obstetric ICU, in the northeast of Brazil, providing the understanding of demographic and obstetric characteristics of hospitalized women who had complications during pregnancy, during delivery, or in the postpartum period. Data are presented from a database collected between October 29, 2018, and September 30, 2019, before the COVID-19 pandemic.

## 2. Methods

### 2.1. Study Design

This research is a cross-sectional study conducted at a public hospital, a referral obstetric centre, located in the city of Recife, state of Pernambuco, northeast region of Brazil: the Instituto de Medicina Integral Prof. Fernando Figueira (IMIP), with data collected between October 29, 2018, and September 30, 2019. This unit is an academic tertiary centre equipped with an obstetric ICU.

### 2.2. Sample of Participants

The study population consisted of pregnant and postpartum women up to the 42nd day after childbirth admitted to the obstetric ICU for any serious clinical, surgical, or obstetric complication for at least 24 h. A database had been previously built on the Research Electronic Data Capture (REDCap) secure web platform to enter data for all ICU patients. This database was used in the external validation study of the CIPHER model in Brazil [20, 21]. Patients previously entered in this database were included in the current study.

### 2.3. Data Collection (Measurement of Results)

We used information on maternal age (years), marital status (with or without partner), body mass index (kg/m^2^), number of prenatal visits, number of pregnancies and parity, reason for admission (direct obstetric, indirect obstetric, and both), time of admission (antepartum or postpartum), gestational age on admission (weeks), length of stay in the ICU (days), early miscarriage, stillbirth, early neonatal death (from birth up to the seventh day of life), type of delivery (vaginal or cesarean), birth weight (g), hospital discharge while pregnant, and number of maternal near-miss and maternal deaths. Women with missing control or demographic variables were included.

### 2.4. Statistical Methods

For statistical analysis, Statistical Package for the Social Sciences (SPSS program) version Statistics 28 was used. For comparison of baseline characteristics of groups with and without the composite outcome, the chi-square test was used for categorical variables, and Fisher's exact test was used for expected values less than 5. It was only possible to use parametric Student's *T*-test for only age and birth weight. For the others, which did not have normal distribution, the test used was the Mann–Whitney *U* test (nonparametric). A significance level of 5% was adopted.

### 2.5. Ethics Approval and Consent to Participate

Institutional Review Board (IRB) approval was obtained at the Instituto de Medicina Integral Prof. Fernando Figueira (IMIP) (CAAE: 52164121.4.0000.5201). This retrospective data collection was considered exempt from requiring a written informed consent.

## 3. Results

**Figure 1:**
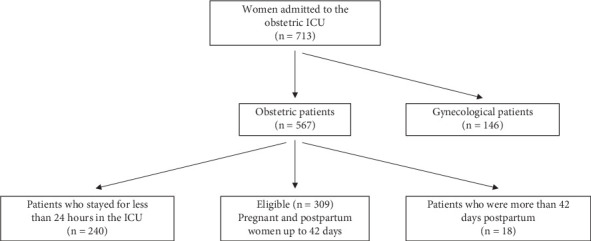
Participant capture flowchart.

Over the study period, 309 women were admitted to the obstetric ICU and considered eligible for the study (Figure 1). Characteristics of the study population are presented in Table 1, comparing women with SMO and with PLTC. Most of the women with SMO were admitted to the ICU for longer and had a shorter gestational period at ICU admission for women admitted antepartum. SMO was observed in 150 (48.5%) women. Of these, 8 (2.6%) were maternal deaths, and 142 (45.9%) had one or more near-miss criteria (Table 2). The condition most associated with SMO was puerperal infection (Table 3).

Causes of maternal death were cerebral haemorrhage after eclampsia, acute pulmonary edema after severe preeclampsia, septic shock after bacterial meningitis, acute pulmonary thromboembolism, peripartum cardiomyopathy, septic shock after systemic infection in an HIV-infected patient, shock in a patient with advanced cervical cancer, and shock after puerperal haemorrhage.

## 4. Discussion

In this study, 150 (48.5%) patients were identified as SMO. However, only 8 (2.6%) women died. In a study conducted in a general ICU in Nigeria [22], the number of maternal deaths was significantly higher compared to the current study (43 from 101 obstetric patients, 42.6%). It is believed that general ICUs adopt different criteria of admission. Obstetric patients admitted to general ICUs tend to have a more severe condition, requiring advanced support such as advanced respiratory support, two or more organ system support, and support for acute reversible failure of another organ system in those with chronic system insufficiency, whereas patients admitted to obstetric ICUs present conscious and have single-organ dysfunction; in fact, obstetric ICUs have incorporated admission criteria from intermediate or high-dependency care units [15]. However, it is possible that many patients admitted to the obstetric ICU were using magnesium sulfate exclusively without any organ dysfunction.

Most women with a SMO were admitted for direct obstetric causes such as hypertensive syndromes, postpartum haemorrhage, and puerperal infection. Also, direct obstetric causes were the most prevalent cause of maternal deaths (5/8). Direct obstetric causes are those resulting from obstetric complications during pregnancy, labour, and puerperium, from interventions, omissions, or incorrect treatment or from a chain of events occurring from any of the above [23]. As a comparison, indirect obstetric causes such as community-acquired pneumonia, acute cardiogenic lung edema, and decompensated diabetes mellitus were less prevalent. Indirect obstetric causes are those resulting from previous existing disease or disease that developed during pregnancy and which was not due to direct obstetric causes but was aggravated by physiologic effects of pregnancy [3, 4]. These findings are in line with data from other studies [5–7], including a study conducted in the southeast of Brazil, which found that direct obstetric causes are the predominant reasons for admission to the ICU and the main causes of maternal death [24].

One or more near-miss criteria were present in 142 (45.9%) women. Near-miss morbidity, which refers to woman who almost died, was more characterised by cardiovascular dysfunction. The use of continuous vasoactive drugs, such as vasodilators and vasoconstrictors, was the most common reason for cardiovascular dysfunction. Vasodilators were administered in severe hypertension and vasoconstrictors in severe hypotension by, for example, septic shock. Other very common criteria were intubation, thrombocytopenia, and hysterectomy.

Puerperal infection was the cause most associated with SMO. Infections and sepsis have historically been linked to maternal deaths and continue to be leading causes of morbidity and mortality among women during and after pregnancy [25]. However, it is noteworthy that maternal death was not observed in these cases, probably because this infection tends to affect a younger, healthier population and, with adequate treatment, has lower mortality rates [26]. Our finding supports previous work by Igbaruma et al. in Nigeria [22], who have argued that infection ranked high in the near-miss morbidity subgroup with reduced mortality rates. Emergency obstetric hysterectomy is recognized as a life-saving intervention responsible for reducing MM related to both haemorrhage and infection [27–29].

Most of the women with SMO were admitted to the ICU for longer and had a shorter gestational period at ICU admission for women admitted antepartum. For obvious reasons, women who had more severe complications needed to stay longer in the ICU. We hypothesize that pregnant women with a shorter gestational period had comorbidities and probably were high-risk pregnancies, thereby increasing the number of severe complications.

### 4.1. Study Limitations

We believe that a larger sample size would demonstrate an association between haemorrhage and SMO. However, this work highlighted the need for a specialised obstetric ICU and aimed to raise awareness about the characteristics of SMO to improve strategies especially related to the prevention of hypertensive syndromes, postpartum haemorrhage, and puerperal infection.

## 5. Conclusion

SMO is a commonly occurring outcome in an obstetric ICU with a great possibility of treatment. The high prevalence of direct obstetric causes such as hypertension, haemorrhage, and infection was relevant in the development of SMO. Puerperal infection was the most significant cause of SMO, reiterating the need for preventive obstetric care during the antepartum, intrapartum, and postpartum periods.

## Figures and Tables

**Table 1:** Characteristics of women admitted to the obstetric intensive care unit. Recife, PE, Brazil, 2018–2019. (*n* = 309).

**Patient characteristics**		**Women with SMO (** **n** = 150**)**	**Women with PLTC (** **n** = 159**)**	**p**
		**Median (IQR) or n (%)**	**Median (IQR) or n (%)**	
Demographics				
Age (years)		28 (21–36)	29 (23–33)	0.55
Marital status^a^	With partner	117 (79.6%)	111 (73%)	0.23
	Without partner	30 (20.4%)	41 (27%)	
BMI (kg/m^2^)^b^		28 (23–30)	30 (26–34)	0.30
Number of prenatal visits^c^	< 6	69 (47.3%)	68 (43.3%)	0.56
	≥ 6	77 (52.7%)	89 (56.7%)	
Number of pregnancies^d^	≤ 2	94 (63.1%)	96 (61.1%)	0.81
	> 2	55 (36.9%)	61 (38.9%)	
Number of births^e^	0	63 (42.3%)	70 (44.3%)	0.80
	≥ 1	86 (57.7%)	88 (55.7%)	
Details of ICU admission				
Reason for admission	Direct obstetric	113 (75.4%)	122 (76.8%)	0.27
	Indirect obstetric	24 (16%)	30 (18.8%)	
	Both	13 (8.6%)	7 (4.4%)	
Timing of admission^f^	Antepartum	29 (21.4%)	38 (26%)	0.45
	Postpartum	106 (78.6%)	108 (74%)	
Gestational age (weeks) at ICU admission (only for women admitted antepartum)		31 (25–34)	32 (29–34)	0.001
Length of ICU stay (days)		5 (4–9)	2 (2–4)	< 0.001
Pregnancy outcomes				
Early pregnancy loss < 22 weeks^g^	Yes	5 (3.5%)	6 (4.3%)	0.99
Livebirth^h^	Yes	113 (86.9%)	114 (87.7%)	> 0.99
	No (stillbirth)	17 (13.1%)	16 (12.3%)	
Early neonatal death^i^	Yes	14 (9.3%)	9 (5.6%)	0.37
Mode of birth^j^	Vaginal	38 (29.2%)	37 (28.7%)	> 0.99
	Cesarean	92 (70.8%)	92 (71.3%)	
Weight at birth (g)^k^		1583 (942–2672)	2235 (1350–2800)	0.50
Still pregnant at hospital discharge^l^	Yes	3 (2.2%)	11 (7.5%)	0.77

*Note:* Missing data, *n* (%): ^a^10 (3.2%); ^b^28 (9.1%); ^c^6 (1.9%); ^d^3 (1%); ^e^2 (0.6%); ^f^28 (9.1%); ^g^26 (8.4%); ^h^49 (15.9%); ^i^52 (16.8%); ^j^50 (16.2%); ^k^53 (17.2%); ^l^28 (9.1%).

Abbreviations: BMI, body mass index; PLTC, potential life-threatening condition; SMO, severe maternal outcome.

**Table 2:** Distribution of causes of maternal near-miss in accordance with the WHO. Recife, PE, Brazil, 2018–2019. (*n* = 309).

**Near-miss criteria**	**n** ^∗^ ** (%)**
Cardiovascular dysfunction	134 (38.4%)
Shock	22 (16.4%)
Cardiorespiratory arrest	10 (7.4%)
Use of vasoactive drugs	80 (59.7%)
Severe hypoperfunsion (lactate > 5 mmol/L)	15 (11.2%)
Severe acidosis (pH < 7.1)	7 (5.3%)
Respiratory dysfunction	71 (20.4%)
Acute cyanosis	3 (4.2%)
Respiratory rate > 40 or < 6	8 (11.2%)
Intubation and nonanesthesia-related AVM	42 (59.1%)
Severe hypoxemia (SpO_2_ < 90% for ≥ 60 min)	6 (8.5%)
PaO2/FiO2 < 200	12 (17%)
Kidney dysfuntion	34 (9.7%)
Oliguria unresponsive to fluids and diuretics	15 (44.1%)
Dialysis for acute renal failure	9 (26.4%)
Acute severe azotemia (creatinine > 3.5 mg/dL)	10 (29.5%)
Hematological or coagulation dysfuntion	48 (13.8%)
Coagulation failure	7 (14.5%)
Massive transfusion of blood or red cells (≥ 5 units)	9 (18.8%)
Severe acute thrombocytopenia (< 50,000 platelets/mL)	32 (66.7%)
Liver dysfunction	13 (3.7%)
Jaundice in the presence of preeclampsia	5 (38.4%)
Severe acute hyperbilirubinemia (bilirubin > 6.0 mg/dL)	8 (61.6%)
Neurological dysfunction	13 (3.7%)
Decreased level of consciousness	8 (61.5%)
Stroke	3 (23.1%)
Uncontrolled status epilepticus	2 (15.4%)
Uterine dysfunction/hysterectomy	36 (10.3%)
Haemorrhage or infection leading to hysterectomy	36 (100%)

⁣^∗^Maternal morbidities presented are not mutually exclusive and include those occurring in women who died.

**Table 3:** Distribution of causes of admission in the obstetric intensive care unit according to maternal outcome. Recife, PE, Brazil, 2018–2019. (*n* = 309).

**Causes—** **n** ** (%)**	**With SMO, ** **n** ** (%)**	**With PLTC, ** **n** ** (%)**	**p**
Hypertensive—165	75 (45.4%)	90 (54.6%)	
Severe preeclampsia	27 (36%)	29 (32.2%)	0.95
Eclampsia	14 (18.6%)	20 (22.2%)	
HELLP syndrome	17 (22.6%)	25 (27.7%)	
Superimposed preeclampsia	16 (21.3%)	14 (15.5%)	
Other	1 (1.3%)	2 (2.2%)	
Haemorrhage—58	30 (51.7%)	28 (48.3%)	
Uterine atony	8 (26.6%)	7 (25%)	0.79
Laceration of the delivery route	0 (0%)	4 (14.2%)	
Placenta previa	0 (0%)	1 (3.5%)	
Placental abruption	4 (13.3%)	5 (17.8%)	
Placental acretism	4 (13.3%)	1 (3.5%)	
Postpartum haemorrhage	11 (36.6%)	6 (21.4%)	
Ruptured ectopic pregnancy	1 (3.3%)	2 (7.1%)	
Other	2 (6.6%)	2 (7.1%)	
Infection—32	20 (62.5%)	12 (37.5%)	
Puerperal infection	7 (35%)	0 (0%)	0.007
Chorioamnionitis	1 (5%)	1 (8.3%)	
Bacterial meningitis	1 (5%)	0 (0%)	
Acute pyelonephritis	3 (15%)	6 (50%)	
Urinary infection	1 (5%)	0 (0%)	
Community-acquired pneumonia	3 (15%)	5 (41.6%)	
Hospital-acquired pneumonia	1 (5%)	0 (0%)	
Viral pneumonia	1 (5%)	0 (0%)	
Other	2 (10%)	0 (0%)	
Hepatic/gastrointestinal tract—6	4 (66.6%)	2 (33.3%)	
Acute fatty liver of pregnancy	3 (75%)	0 (0%)	
Other	1 (25%)	2 (100%)	
Cardiologic/pulmonary—24	12 (50%)	12 (50%)	
Peripartum cardiomyopathy	1 (8.3%)	0 (0%)	0.24
Congestive heart failure	1 (8.3%)	0 (0%)	
Acute cardiogenic lung edema	3 (25%)	0 (0%)	
Valve disease	1 (8.3%)	3 (25%)	
Acute pulmonary thromboembolism	1 (8.3%)	3 (25%)	
Acute chest syndrome	1 (8.3%)	0 (0%)	
Other	4 (33.3%)	6 (50%)	
Renal/metabolic/endocrinological*—*12	5 (41.6%)	7 (58.4%)	
Acute kidney injury or acute kidney failure or acute chronic kidney failure	3 (60%)	0 (0%)	
Decompensated diabetes mellitus	1 (20%)	6 (85.7%)	
Diabetic ketoacidosis	1 (20%)	0 (0%)	
Other	0 (0%)	1 (14.3%)	
Hematological—9	4 (44.4%)	5 (55.6%)	
Sickle cell anemia	0 (0%)	2 (40%)	0.61
Sickling crisis	1 (25%)	1 (20%)	
TTP	1 (25%)	0 (0%)	
Other	2 (50%)	2 (40%)	
Central nervous system—6	3 (50%)	3 (50%)	
Epilepsy or seizure	1 (33.3%)	3 (100%)	0.61
Other	2 (66.7%)	0 (0%)	
Total	150	159	

Abbreviations: HELLP, hemolysis, elevated liver enzymes, low platelet; PLTC, potential life-threatening condition; SMO, severe maternal outcome; TTP, thrombotic thrombocytopenic purpura.

## Data Availability

Data are available on request—Flávio Xavier da Silva, Faculdade Pernambucana de Saúde, 4861 Avenida Mal. Mascarenhas de Morais, Imbiribeira, Recife, PE, Brazil. 51150000; E-mail: flavioxavier@fps.edu.br.

## References

[1] Souza J. P., Cecatti J. G., Haddad S. M. (2012). The WHO Maternal Near-Miss Approach and the Maternal Severity Index Model (MSI): Tools for Assessing the Management of Severe Maternal Morbidity. *PLoS One*.

[2] Say L., Souza J. P., Pattinson R. C., WHO working group on Maternal Mortality and Morbidity classifications (2009). Maternal Near Miss -- Towards a Standard Tool for Monitoring Quality of Maternal Health Care. *Best Practice & Research Clinical Obstetrics & Gynaecology*.

[3] Adeniran A. S., Ocheke A. N., Nwachukwu D. (2019). Non-Obstetric Causes of Severe Maternal Complications: A Secondary Analysis of the Nigeria Near-Miss and Maternal Death Survey. *BJOG*.

[4] Silva F. X., Katz L., Cecatti J. G. (2023). Prognostic Scores for Prediction of Maternal Near Miss and Maternal Death After Admission to an Intensive Care Unit: A Narrative Review. *Health Care for Women International*.

[5] Adeoye I. A., Ijarotimi O. O., Fatusi A. O. (2015). What Are the Factors That Interplay From Normal Pregnancy to Near Miss Maternal Morbidity in a Nigerian Tertiary Health Care Facility?. *Health Care for Women International*.

[6] Chowdhury M. E., Ahmed A., Kalim N., Koblinsky M. (2009). Causes of Maternal Mortality Decline in Matlab, Bangladesh. *Journal of Health, Population and Nutrition*.

[7] Koblinsky M., Chowdhury M. E., Moran A., Ronsmans C. (2012). Maternal Morbidity and Disability and Their Consequences: Neglected Agenda in Maternal Health. *Journal of Health, Population and Nutrition*.

[8] World Health Organization (2023). *Trends in Maternal Mortality 2000 to 2020: Estimates by WHO*.

[9] World Health Organization (2024). *Maternal Mortality*.

[10] Knight M., Bunch K., Fleker A. (2023). *Saving Lives. Improving Mothers’ Care Core Report–Lessons Learned to Inform Maternity Care From the UK and Ireland Confidential Enquiries Into Maternal Deaths and Morbidity 2019-21*.

[11] Maine D. (1991). *Safe Motherhood Programs: Options and Issues*.

[12] World Health Organization (2011). *Evaluating the Quality of Care for Severy Pregnancy Complications: The WHO Near-Miss Approach for Maternal Health*.

[13] Thaddeus S., Maine D. (1994). Too Far to Walk: Maternal Mortality in Context. *Social Science & Medicine*.

[14] Soares F. M., Pacagnella R. C., Tunçalp Ö. (2020). Provision of Intensive Care to Severely Ill Pregnant Women Is Associated With Reduced Mortality: Results From the WHO Multicountry Survey on Maternal and Newborn Health. *International Journal of Gynecology & Obstetrics*.

[15] Zeeman G. G. (2006). Obstetric Critical Care: A Blueprint for Improved Outcomes. *Critical Care Medicine*.

[16] Rulisa S., Umuziranenge I., Small M., van Roosmalen J. (2015). Maternal Near Miss and Mortality in a Tertiary Care Hospital in Rwanda. *BMC Pregnancy and Childbirth*.

[17] You W. B., Chandrasekaran S., Sullivan J., Grobman W. (2013). Validation of a Scoring System to Identify Women With Near-Miss Maternal Morbidity. *American Journal of Perinatology*.

[18] Health Ministry of Brazil *DATASUS: Department of Informatics of the SUS. Health Information/Vital Statistics*.

[19] Health Ministry of Brazil (2021). COVID-19 Observatory. COVID-19 and Maternal Mortality. COVID-19 Observatory Bulletin. https://portal.fiocruz.br/observatorio-covid-19.

[20] Silva F. X., Parpinelli M. A., Oliveira-Neto A. F. (2022). Prognostic Value of an Estimate-of-Risk Model in Critically Ill Obstetric Patients in Brazil. *Obstetrics & Gynecology*.

[21] Silva F. X., Parpinelli M. A., Oliveira-Neto A. F. (2022). Comparison of the CIPHER Prognostic Model With the Existing Scores in Predicting Severe Maternal Outcomes During Intensive Care Unit Admission. *International Journal of Gynaecology and Obstetrics*.

[22] Igbaruma S., Olagbuji B., Aderoba A., Kubeyinje W., Ande B., Imarengiaye C. (2016). Severe Maternal Morbidity in a General Intensive Care Unit in Nigeria: Clinical Profiles and Outcomes. *International Journal of Obstetric Anesthesia*.

[23] World Health Organization (2009). Averting Maternal Death and Disability (AMDD). *Monitoring Emergency Obstetric Care: A Handbook*.

[24] Andrade M. S., Bonifácio L. P., Sanchez J. A. C. (2022). Fatores Associados à Morbidade Materna Grave em Ribeirão Preto, São Paulo, Brasil: Estudo de Corte Transversal. *Reports in Public Health*.

[25] Liu P., Zhang X., Wang X. (2023). Maternal Sepsis in Pregnancy and the Puerperal Periods: A Cross-Sectional Study. *Frontiers in Medicine*.

[26] Barton J. R., Sibai B. M. (2012). Severe Sepsis and Septic Shock in Pregnancy. *Obstetrics & Gynecology*.

[27] Zhang Y., Yan J., Han Q. (2017). Emergency Obstetric Hysterectomy for Life-Threatening Postpartum Hemorrhage: A 12-Year Review. *Medicine (Baltimore)*.

[28] Chawla J., Arora C. D., Paul M., Ajmani S. N. (2015). Emergency Obstetric Hysterectomy: A Retrospective Study From a Teaching Hospital in North India Over Eight Years. *Oman Medical Journal*.

[29] Daskalakis G., Anastasakis E., Papantoniou N., Mesogitis S., Theodora M., Antsaklis A. (2007). Emergency Obstetric Hysterectomy. *Acta Obstetricia et Gynecologica Scandinavica*.

